# Case Report: Transient immune dysregulation in early infancy mimicking severe systemic autoimmunity with complete spontaneous resolution

**DOI:** 10.3389/fimmu.2026.1894076

**Published:** 2026-07-22

**Authors:** Hana Matković, Maja Ban, Ivanka Kos, Maša Davidović, Kristina Vrljičak, Ana Kozmar, Mirta Lamot, Zoran Miovski, Alessandra Tesser, Alberto Tommasini, Andrea Taddio, Lovro Lamot

**Affiliations:** 1 Division of Pediatric Nephrology, Dialysis and Transplantation, Department of Pediatrics, University Hospital Centre Zagreb, Zagreb, Croatia; 2 Department of Laboratory Diagnostics, University Hospital Centre Zagreb, Zagreb, Croatia; 3 Faculty of Pharmacy and Biochemistry, University of Zagreb, Zagreb, Croatia; 4 Division of Neonatology, Department of Gynecology and Obstetrics, Sestre milosrdnice University Hospital Center, Zagreb, Croatia; 5 Clinic for Cardiovascular Diseases, University Hospital Centre Rijeka, Rijeka, Croatia; 6 Institute for Maternal and Child Health, IRCCS Burlo Garofolo, Trieste, Italy; 7 Institute for Maternal and Child Health, IRCCS Burlo Garofolo and University of Trieste, Trieste, Italy; 8 Department of Pediatrics, University of Zagreb School of Medicine, Zagreb, Croatia

**Keywords:** antinuclear antibodies (ANA), autoimmunity, child, immune development, immune dysregulation, immune tolerance, infant

## Abstract

Early infancy represents a critical window of immune system maturation during which transient dysregulation may result in atypical inflammatory or autoimmune-like phenotypes. Distinguishing such self-limited processes from true systemic autoimmune disease remains a major clinical challenge. We report an infant presenting with prominent cutaneous manifestations, markedly elevated inflammatory markers, hypocomplementemia, and broad high-titer autoantibody positivity including antinuclear, anti-dsDNA, extractable nuclear antigen, and antineutrophil cytoplasmic antibodies. Despite a laboratory profile strongly suggestive of systemic autoimmune disease, the patient remained clinically stable without evidence of organ involvement, including renal, neurological, or cardiopulmonary systems. Immunophenotyping demonstrated lymphocytosis with expansion of CD19+ B cells, while T-cell subsets and natural killer cells remained within expected ranges. Interferon signature analysis was negative, and whole-exome sequencing did not identify a causative monogenic disorder. All immunological abnormalities resolved spontaneously without immunosuppressive therapy. During follow-up, the patient developed transient neutropenia and atopic dermatitis, both with a self-limited course. This case highlights a transient systemic immune phenotype characterized by expansion of the CD19+ B-cell compartment, broad humoral immune activation and complement consumption in early infancy, followed by complete restoration of immune homeostasis. Recognition of such self-limited immune dysregulation is essential to avoid unnecessary immunosuppressive therapy and provides insight into mechanisms of immune tolerance during early human development.

## 1 Introduction

Early life represents a uniquely dynamic period of immune system development, during which the balance between immune activation and tolerance is continuously shaped by intrinsic maturation and environmental exposure (1, 2). The neonatal and early infant immune system is characterized by functional immaturity of adaptive responses, evolving regulatory networks, and progressive postnatal immune education driven by environmental antigens and commensal microbiota (3). This developmental plasticity is essential for the establishment of immune homeostasis but also renders the immune system susceptible to transient dysregulation (4).

In clinical practice, the detection of hypocomplementemia together with high-titer autoantibodies is generally interpreted as evidence of systemic autoimmune disease or monogenic immune dysregulation (5). This interpretation is biologically grounded, as persistent immune activation, impaired clearance of apoptotic material, complement pathway abnormalities, and dysregulated innate immune signaling are central mechanisms in diseases such as systemic lupus erythematosus and related immune-mediated conditions (6). In addition, interferon-driven inflammatory pathways play a key role in a subset of monogenic and acquired immune disorders, further reinforcing the association between such laboratory findings and severe disease (7). Consequently, these abnormalities typically trigger extensive diagnostic evaluation and often prompt early consideration of immunosuppressive therapy due to concern for progressive organ involvement (8, 9).

Yet, the interpretation of immunological findings in early infancy remains particularly challenging (10). Reference intervals are age-dependent, immune responses are still evolving, and laboratory abnormalities may not carry the same diagnostic significance as in older children or adults (2). This is especially relevant for autoantibody testing in pediatrics, where antinuclear antibody positivity may be detected even in otherwise healthy children and requires careful clinical contextualization (11–13). While such findings are most often low-titer and transient, their presence can complicate clinical decision-making, particularly when accompanied by additional abnormalities such as complement consumption (14, 15). Against this background, the occurrence of broad high-titer autoantibody positivity combined with hypocomplementemia remains highly concerning and is usually interpreted as indicative of severe immune-mediated disease (16, 17). However, the extent to which such findings may, in rare circumstances, reflect a transient and self-limited disturbance of immune regulation rather than established pathology is not well defined.

Here, we describe an infant presenting with a constellation of immunological abnormalities closely mimicking systemic autoimmune disease, yet without organ involvement and with complete spontaneous resolution. This case provides a unique opportunity to reconsider the interpretation of severe immunological abnormalities in early infancy and to explore mechanisms of transient immune dysregulation and restoration of immune tolerance.

## 2 Case description

A previously healthy male infant, born at term after an uncomplicated pregnancy and delivery, presented at the age of 6 weeks with acute onset of a generalized cutaneous eruption and edema of the right hand, prompting hospital admission for further evaluation. The infant had normal prenatal history, appropriate birth weight (3880 g), and normal early postnatal adaptation. Family history was negative for autoimmune or renal diseases. Prior to this admission, at 2 weeks of age, the patient had been hospitalized abroad for a similar episode of edema and rash, initially interpreted as acute hemorrhagic edema of infancy. At that time, he received short-course antibiotic therapy, although infectious etiology was not confirmed.

At presentation, the infant was in excellent general condition, afebrile, with preserved feeding, normal growth, and no systemic symptoms. Physical examination revealed erythematous, partially blanching maculopapular and urticarial-like lesions distributed over the extremities and trunk, along with localized edema of the right hand (**Figure 1**). No lymphadenopathy, hepatosplenomegaly, or neurological abnormalities were noted. Initial laboratory evaluation demonstrated elevated inflammatory markers, including erythrocyte sedimentation rate (up to 73–100 mm/h) and mildly increased C-reactive protein (up to 13 mg/L), suggesting systemic inflammation (**Figure 2**). Microbiological investigations, including extended respiratory and gastrointestinal viral panels, TORCH screening, urine PCR testing for CMV, as well as repeated CMV and EBV serology, were negative, excluding infectious triggers. Immunological testing revealed profound hypocomplementemia, with markedly reduced complement levels (C3 as low as 0.55–0.57 g/L, C4 <0.03 g/L, and CH50 activity reduced to 2%), consistent with complement consumption. Concurrently, extensive autoantibody profiling demonstrated high-titer positivity, including antinuclear antibodies (ANA, titer up to 1:800), anti-dsDNA antibodies (up to 60 IU/mL), and multiple extractable nuclear antigen antibodies, including anti-SSA/Ro52, anti-U1-RNP, anti-PM-Scl, and anti-PCNA (**Table 1**). However, confirmatory testing using Crithidia luciliae immunofluorescence (CLIFT) was negative, indicating the absence of high-affinity anti-dsDNA antibodies. Additionally, antineutrophil cytoplasmic antibodies were detected (p-ANCA and atypical ANCA, titers up to 1:160), with positive anti-PR3 and anti-MPO antibodies. Maternal autoimmune evaluation was performed immediately after the detection of autoantibody positivity in the infant and included ANA, anti-dsDNA, ENA panel antibodies, ANCA, antiphospholipid antibodies, lupus anticoagulant, and complement studies. All autoantibody tests were negative, while C3, C4, and CH50 levels were within normal ranges, making passive transplacental transfer of maternal autoantibodies highly unlikely.

**Figure 1:**
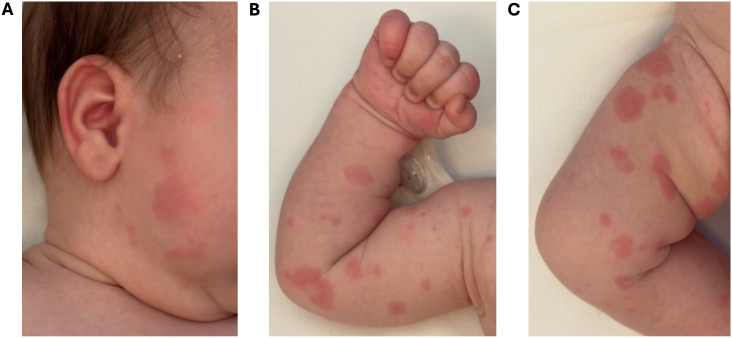
Cutaneous manifestations at presentation. Generalized erythematous maculopapular and urticarial-like lesions involving the face **(A)**, upper **(B)** and lower **(C)** extremities and trunk (not shown) in a 6-week-old infant at disease onset. Lesions were partially blanching and non-necrotic, without purpura or ulceration, and were not associated with systemic symptoms.

**Figure 2:**
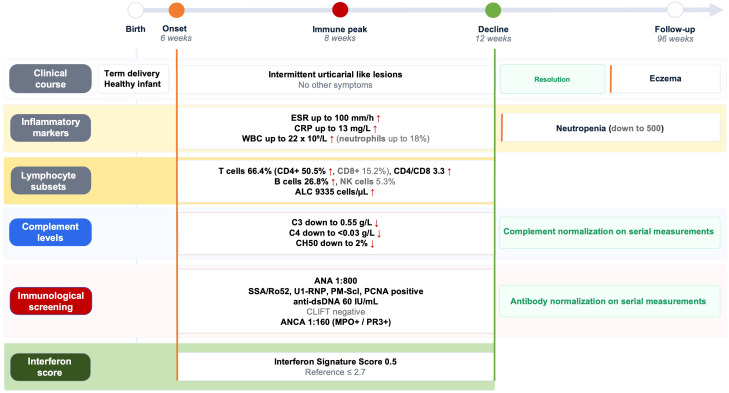
Timeline of clinical course and immunological findings. Schematic representation of the disease course from birth to 96 weeks. Onset at 6 weeks was marked by urticarial-like lesions without systemic involvement, with peak immunological abnormalities at 8 weeks and spontaneous resolution by 12 weeks. Laboratory findings included elevated inflammatory markers, lymphocytosis with CD19+ B-cell expansion, hypocomplementemia (low C3, C4, CH50), and broad autoantibody positivity (ANA, anti-dsDNA, ENA, ANCA). Interferon signature was normal. All abnormalities normalized on serial measurements without immunosuppressive therapy. Follow-up showed transient neutropenia and mild eczema.

**Table 1:** Immunological and complement profile at peak disease activity.

Parameter	Result	Reference range	Interpretation
Complement system			
C3	0.57 g/L	0.90–1.80	Decreased
C4	<0.03 g/L	0.10–0.40	Markedly decreased
CH50 (classical pathway activity)	2%	69–129%	Severely reduced
Antinuclear antibodies (ANA)			
ANA titer	1:800	<1:100	Strongly positive
ANA pattern	AC-4* + AC-5* +	—	Fine and coarse granular nuclear pattern
Anti-dsDNA	60 IU/mL	<30 negative	Positive
CLIFT (Crithidia luciliae)	Negative	<1:100	Negative
Nuclear autoantibodies (ENA and related)			
Anti-histone antibodies	Negative	<30 negative	Negative
Anti-SSA (Ro52)	96 AU/mL	<30 negative	Positive
Anti-U1-RNP	64 AU/mL	<30 negative	Positive
Anti-PM-Scl	47 AU/mL	<30 negative	Positive
Anti-PCNA	68 AU/mL	<30 negative	Positive
Anti-SSB	Negative	<30 negative	Negative
Anti-Sm	Negative	<30 negative	Negative
Anti-Scl-70	Negative	<30 negative	Negative
Anti-Jo-1	Negative	<30 negative	Negative
Anti-CENP-B	Negative	<30 negative	Negative
Anti-ribosomal antibodies	Negative	<30 negative	Negative
ANCA-associated antibodies			
ANCA (IIF)	1:160	<1:20	Positive
Anti-MPO	50 CU	<20	Positive
Anti-PR3	26.7 CU	<20	Borderline positive
Antiphospholipid antibodies and related tests			
Anticardiolipin IgG	18.1 CU	<20	Negative
Anticardiolipin IgM	1.8 CU	<20	Negative
Lupus anticoagulant	Negative	Negative	Negative

Summary of complement parameters and autoantibody findings obtained at the time of maximal immunological abnormalities. Results demonstrate marked hypocomplementemia with reduced C3, C4, and CH50, accompanied by broad high-titer autoantibody positivity, including ANA, anti-dsDNA, multiple extractable nuclear antigen antibodies, and ANCA-associated antibodies. CLIFT assay was negative, indicating absence of high-affinity anti-dsDNA antibodies. Antiphospholipid antibodies and lupus anticoagulant were negative.

*International Consensus on ANA nomenclature (ICAP).

Despite this striking serological profile suggestive of systemic autoimmune disease, the patient remained clinically stable with no evidence of organ involvement. Renal function tests, including serum creatinine and urea, were within normal limits, and urinalysis showed no proteinuria or hematuria. Cardiac evaluation, including electrocardiography and echocardiography, demonstrated normal cardiac structure and function. Neurological assessment, including cranial ultrasonography and electroencephalography, revealed no abnormalities. Abdominal ultrasonography showed normal kidneys and no organomegaly. Skin biopsy of lesional tissue demonstrated a dense perivascular and interstitial inflammatory infiltrate rich in eosinophils extending into the subcutis, without evidence of fibrin deposition or leukocytoclastic vasculitis. The findings were atypical for urticaria and raised the possibility of eosinophil-driven inflammatory processes, including hypereosinophilic conditions.

Given the discordance between extensive immunological abnormalities and benign clinical status, a comprehensive diagnostic work-up was performed to exclude monogenic immune dysregulation disorders (**Figure 2**). Interferon signature analysis revealed a normal interferon score (0.5; reference ≤2.7), arguing against interferonopathies. Whole-exome sequencing (NGS-based analysis of 603 immunology-related genes) did not identify pathogenic or likely pathogenic variants associated with the clinical phenotype. A heterozygous variant in the *PIEZO1* gene (c.7427G>A; p.Arg2476His) was detected and classified as a variant of uncertain significance (VUS), with low population frequency (gnomAD 1.54×10⁻^5^) and predicted potential functional impact, although its clinical relevance remained unclear. Immunophenotyping of peripheral blood demonstrated leukocyte counts within age-appropriate ranges (12.7 ×10^9^/L), with lymphocyte predominance (73.5%) and elevated absolute lymphocyte count (9335 cells/µL). A notable expansion of CD19+ B lymphocytes was observed (26.8%; 2502 cells/µL), exceeding reference values, while T-cell subsets (CD3+, CD4+, CD8+) and NK cells were within normal limits, with preserved CD4/CD8 ratio (3.3). These findings supported expansion of the CD19+ B-cell compartment in the absence of features suggestive of lymphoproliferative disease.

During hospitalization, no immunosuppressive or immunomodulatory therapy was initiated. The patient was managed conservatively, including dietary modification with a semi-elemental formula due to suspected cow’s milk protein allergy. Over the course of follow-up, a progressive and spontaneous resolution of immunological abnormalities was observed. Complement levels normalized, and previously positive autoantibodies, including ANA, anti-dsDNA, ENA panel, and ANCA, became negative or borderline on repeated testing after six months. No clinical signs of systemic autoimmune disease emerged.

In the subsequent months, the patient developed mild atopic dermatitis and intermittent neutropenia. Absolute neutrophil counts fluctuated, with nadir values around 0.67–1.86 ×10^9^/L, without associated severe infections. Immunohematological testing revealed circulating anti-leukocyte IgM antibodies, although these findings were nonspecific and not diagnostic of autoimmune neutropenia. The dermatological manifestations evolved into mild eczema responsive to topical therapy. Suspected cow’s milk protein allergy was managed with dietary adjustments, with good clinical tolerance and no confirmed IgE-mediated sensitization on skin prick testing. Throughout longitudinal follow-up, the child remained in good general condition, with normal growth and neurodevelopment. Importantly, no recurrence of systemic inflammation, no organ involvement, and no progression toward a defined autoimmune or autoinflammatory disorder were observed.

## 3 Discussion

This case illustrates a profound yet reversible disturbance of immune homeostasis in early infancy, presenting as a systemic immune phenotype that closely mimicked severe autoimmune disease. The defining feature of this presentation lies in the marked mismatch between the severity of immunological abnormalities and the benign clinical course. At presentation, the combination of hypocomplementemia and broad high-titer autoantibody positivity strongly suggested immune complex–mediated disease, as described in systemic lupus erythematosus and related disorders (5, 6, 16, 17). The complete normalization of both complement levels and autoantibody profiles in this patient, in the absence of treatment, argues against stable autoimmune pathology and supports a fundamentally different mechanism.

The B-cell compartment provides the first key element for mechanistic interpretation. Expansion of CD19+ B cells occurred in the setting of preserved T-cell subsets and normal natural killer cell counts, a pattern suggestive of broad humoral immune activation. In early infancy, the B-cell repertoire is enriched in autoreactive specificities because central and peripheral tolerance checkpoints are not yet fully mature (18–20). Under conditions of immune stimulation, this developmental state may permit transient expansion of autoreactive clones, resulting in broad autoantibody production. The observed autoantibody profile, including ANA, anti-dsDNA, ENA, and ANCA, is therefore best interpreted as a reflection of global B-cell activation rather than antigen-driven immune responses (18, 19, 21).

This interpretation is closely linked to the maturation of immune tolerance. During infancy, regulatory pathways that normally constrain autoreactive lymphocytes are still developing, while postnatal immune adaptation continues under the influence of environmental exposure and tissue-specific immune education (1, 2, 4). Central tolerance in the bone marrow is not yet fully efficient, and peripheral tolerance, including regulatory T-cell function, may be insufficient to fully suppress transient autoreactivity (22, 23). In this context, a temporary disruption of tolerance mechanisms may allow expansion of autoreactive B-cell populations. The subsequent normalization of immunological findings suggests re-establishment of immune regulation, with contraction of autoreactive clones and restoration of homeostasis. Nevertheless, detailed characterization of B-cell subsets, naïve and memory T-cell compartments, and regulatory T-cell populations was not available in the present case. Consequently, the proposed mechanisms of transient immune dysregulation and restoration of immune tolerance should be regarded as hypothesis-generating rather than definitive. The observed pattern of reduced C3, C4, and CH50 is consistent with complement consumption, likely reflecting transient immune complex formation during this phase of immune dysregulation (5, 17).

An additional aspect of the clinical phenotype that warrants consideration is the eosinophil-rich infiltrate identified in the skin biopsy. Although the findings were not consistent with vasculitis or immune complex-mediated tissue injury, they raise the possibility of concurrent allergic or eosinophilic inflammatory mechanisms. This interpretation is supported by the later development of mild atopic dermatitis and the clinical suspicion of cow’s milk protein allergy. However, no peripheral eosinophilia was present during the acute phase, and isolated allergic or eosinophilic disorders would not readily explain the profound hypocomplementemia, markedly reduced CH50 activity, and broad autoantibody positivity seen in this patient. Therefore, eosinophilic inflammation may have contributed to part of the clinical phenotype but is unlikely to represent its sole underlying mechanism.

Innate immune activation likely represents an additional layer of this process. Early life is characterized by distinct innate immune responsiveness, including heightened sensitivity to pattern recognition receptor signaling and age-specific inflammatory set points (1–3). While microbiological investigations remained negative, this does not exclude transient innate stimulation. One limitation of the present case is that Parvovirus B19 infection could not be fully excluded. Testing was specifically requested during the diagnostic evaluation because of the virus’ known association with transient autoimmune-like manifestations and autoantibody production. However, the assay was unavailable at the time and no final result was obtained. Subclinical or rapidly cleared environmental triggers may therefore have provided sufficient signals to drive expansion of the CD19+ B-cell compartment. The absence of a type I interferon signature further supports this interpretation and argues against interferon-mediated disease, suggesting involvement of alternative innate immune pathways rather than a classical interferonopathy (7, 9, 24).

A heterozygous variant in the *PIEZO1* gene (c.7427G>A; p.Arg2476His) was identified and classified as a variant of uncertain significance. *PIEZO1* encodes a mechanosensitive ion channel with emerging roles in immune regulation, including modulation of innate immune responses and inflammatory signaling (25). Recent studies have suggested that PIEZO1-mediated mechanotransduction may influence immune cell activation and cytokine production (25). Although the functional impact of the identified variant remains unknown, it is conceivable that altered PIEZO1 signaling could modulate immune responsiveness during early life. However, given its classification as a variant of uncertain significance and the absence of functional validation, any causal relationship between this variant and the observed immune phenotype remains speculative.

Several lines of evidence argue against systemic autoimmune disease. Despite extensive serological abnormalities, there was no evidence of renal, neurological, or cardiopulmonary involvement. CLIFT testing remained negative, arguing against the presence of CLIFT-detectable high-avidity anti-dsDNA antibodies typically associated with immune complex–mediated disease. In addition, the absence of an interferon signature further reduces the likelihood of interferon-driven immune pathology (7, 9, 24). Most importantly, all abnormalities resolved without immunosuppressive therapy. This clinical course is not consistent with established autoimmune disease, which is typically characterized by persistence or progression of immune dysregulation and by clinically relevant organ disease over time (6, 26, 27).

The subsequent clinical course is consistent with dynamic immune remodeling. The later occurrence of transient neutropenia and mild atopic dermatitis may reflect ongoing maturation of the immune system rather than emergence of a distinct pathological process. Early-life immune perturbations are known to influence subsequent immune trajectories, including transient cytopenias and atopic manifestations (28, 29). In this context, these findings may represent a continuation of the same adaptive process.

From a clinical perspective, this case underscores the need for cautious interpretation of immunological findings in early infancy. Age-dependent immune maturation and variability in pediatric reference intervals complicate interpretation, particularly for autoantibody testing in otherwise clinically stable infants (10–13). Laboratory abnormalities may not directly correspond to clinically relevant disease. In the absence of major organ involvement, careful monitoring may represent an appropriate management strategy, even in the presence of striking serological abnormalities (30). Such an approach may help avoid unnecessary exposure to immunosuppressive therapy while preserving diagnostic vigilance. Taken together, these observations support the hypothesis of a transient, self-limited immune phenotype in early infancy, characterized by expansion of the CD19+ B-cell compartment, temporary disruption of immune tolerance, and innate immune–driven amplification, followed by restoration of immune homeostasis.

## 4 Patient perspective

The patient’s parents described the diagnostic process as emotionally demanding, primarily because of the discrepancy between the child’s good general condition and the severity of the laboratory abnormalities. The possibility of systemic autoimmune disease and the potential need for long-term immunosuppressive therapy were a major source of concern. Although the child remained clinically stable, the interpretation of the immunological findings was difficult for the family to understand. Over time, the spontaneous normalization of all abnormalities without specific treatment was experienced as reassuring. In retrospect, the course was perceived as unexpected but ultimately favorable, and the parents expressed appreciation that a conservative, observation-based approach was adopted.

## Data Availability

The original contributions presented in the study are included in the article/supplementary material. Further inquiries can be directed to the corresponding author.
